# An open-source BLDC motor test bed utilizing ESC telemetry for cost-effective educational laboratories

**DOI:** 10.1016/j.ohx.2026.e00799

**Published:** 2026-05-30

**Authors:** Uun Triyas Yuni Kurniawan, Faridah, Sunarno

**Affiliations:** Master Program of Engineering Physics, Department of Nuclear Engineering and Engineering Physics, Faculty of Engineering, Universitas Gadjah Mada, Jl. Grafika No. 2, Yogyakarta 55281, Indonesia

**Keywords:** BLDC motor, ESC telemetry, ESP32, Low-cost dynamometer, Open-source hardware, Engineering education

## Abstract

Hands-on motor characterization is essential in engineering education, but commercial dynamometers often exceed USD 15,000. This paper presents an open-source BLDC motor test bed that reduces cost by combining off-the-shelf e-bike components, ESC-native telemetry, an ESP32 controller, and a floating-caliper torque measurement mechanism. The Votol EM-50 ESC provides voltage, current, speed, and temperature telemetry, reducing the need for external electrical instrumentation, while a low-cost load cell and HX711 amplifier measure braking torque through a calibrated lever arm. Beyond hardware implementation, the platform integrates experimental validation with a dq-axis-based BLDC motor model to support interpretation of speed response, torque estimation, and transmission losses. Validation showed electrical measurement errors below 3%, torque error below 5%, expanded torque uncertainty below 0.5%, and model-versus-experiment speed-response agreement with an RMSE of 14.8 rpm. The complete system costs USD 370–488 and is supported by open firmware, wiring diagrams, bill of materials, experimental data, assembly documentation, and supplementary videos archived on Zenodo and GitHub.

## Graphical abstract

**Figure:**
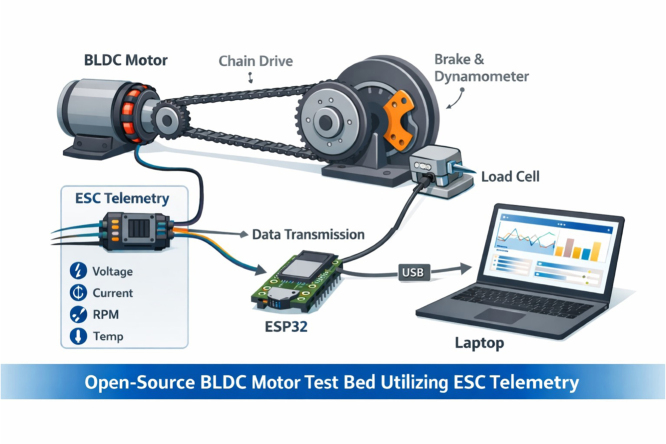

## Specifications table

**Table:** 

Hardware name	Open-Source BLDC Motor Characterization Platform
Subject area	• Engineering • Materials Science • Education

Hardware type	• Measurement of physical properties • Laboratory sensors and instrumentation

Closest commercial analog	Magtrol DSP6001 Dynamometer ($15,000+)
Open source license	CERN Open Hardware Licence Version 2 – Strongly Reciprocal
Cost of hardware	USD 370–488, depending on regional pricing

Source file repository	Zenodo archive: https://doi.org/10.5281/zenodo.20035899; GitHub repository: https://github.com/uun3406/BLDC-TestBed-HardwareX

## 1 Hardware in context

### Motivation.

Laboratory courses on electric machines are essential for engineering students, yet the equipment needed for motor characterization – dynamometers – is often prohibitively expensive. A typical commercial system like the Magtrol DSP6001 costs over $15,000, putting it out of reach for many institutions, especially in developing countries [1]. This financial barrier means students miss the chance to directly observe torque-speed curves, efficiency maps, and transient responses, leaving a gap between theory and practice.

### Deconstructing the cost barrier.

The high price of commercial dynamometers is not a single lump sum but the result of several design choices that drive up complexity and component costs. Identifying these drivers is the first step toward an affordable alternative:

1. **Dedicated torque transducers:** Precision inline rotary sensors demand expensive materials, tight machining tolerances, and factory calibration. Non-contact rotary torque sensors with encoder feedback, typical for test benches, are priced between $3998 and $6000 [2], [3]. Even simpler reaction-type torque sensors cost upwards of $1698 [4]. Moreover, ISO 17025 calibration services for such sensors add $300–$900 per cycle [5].
2. **External electrical instrumentation:** Separate voltage dividers, current sensors, and power analyzers easily add $200–$500, not to mention the wiring and calibration effort. For perspective, a Fluke 87V multimeter with NIST-traceable calibration is $564–$692, and a current clamp (i400) is $178–$218 [6]. Even an entry-level DAQ like the USB-6001 lists at $351 from authorized distributors, with official pricing often quote-based [7]. Low-cost DAQs such as the USB-600x series also lack user calibration flexibility and official calibration services [8].
3. **Proprietary data acquisition systems:** Custom DAQ hardware paired with closed-source software creates vendor lock-in and significant expense. LabVIEW Professional, widely used in motor testing, is priced at $2750 for a yearly subscription or $9625 for a perpetual license [9]. Vendor brochures confirm that motor testing software is typically sold separately from the dynamometer hardware [10].
4. **Specialized mechanical fabrication:** Precision-machined frames, custom couplings, thermal management, and safety enclosures add $300–$800. Commercial systems offer advanced features such as full motor ramp testing and integrated cooling, which require complex mechanical design [3].

Beyond the initial purchase, recurring costs for calibration and maintenance add up. Torque sensor calibration under ISO 17025 can cost $300–$900 per cycle [5], and DAQ recalibration adds further overhead. These hidden expenses make total ownership significantly higher than the sticker price.

### Prior open-source efforts and their limitations.

Several open-source dynamometers have been proposed to lower costs [11], [12]. While inspiring, many still depend on expensive torque transducers or require CNC machining, 3D printing, or specialized skills not available in all educational settings. Few take advantage of the built-in sensing capabilities of modern motor controllers, and most add external sensors that increase both cost and complexity.

### The opportunity in e-bike technology.

The rapid growth of electric vehicles has made high-power BLDC motors and sophisticated controllers remarkably affordable. Modern e-bike controllers, such as the Votol EM-50 series, continuously monitor voltage, current, temperature, and speed for their own operation—data that is streamed via serial telemetry at 9600 baud [13], [14]. Educational projects typically overlook this embedded sensing capability and instead add external sensors. Meanwhile, low-cost microcontrollers like the ESP32 (priced at about $5) can easily capture and process this telemetry [15], [16].

### Contributions.

This work makes three primary contributions that directly counter the cost drivers identified above:

1. **Elimination of external electrical instrumentation** by decoding ESC telemetry (voltage, current, speed, temperature), drastically reducing cost and wiring complexity.
2. **Replacement of precision torque transducers** with a floating-caliper mechanism using a $3 load cell and a simple lever arm, providing educational-grade torque measurement.
3. **A mechanically simple and globally reproducible architecture** built from off-the-shelf e-bike components and basic welding, with all design files openly archived.

### Distinct scientific contribution compared with prior open-source dynamometers.

While several low-cost dynamometer platforms have been reported, most rely on external electrical instrumentation and direct torque sensing architectures. In contrast, the proposed system introduces a different integration strategy by combining ESC-native telemetry acquisition with a physics-informed system model. This integration enables (i) elimination of external electrical sensors, (ii) indirect torque estimation using DC bus current with experimentally bounded error, and (iii) a unified framework linking experimental measurements with dq-axis-based motor modeling. To the authors’ knowledge, this combination has not been explicitly demonstrated in prior open-source BLDC test beds intended for teaching laboratories.

### Scientific contribution beyond cost reduction.

Beyond cost reduction, this work contributes a validated framework that integrates low-cost telemetry-based measurement with physics-based system modeling. Unlike conventional approaches that rely on direct phase current measurement, this system demonstrates that DC bus current from ESC telemetry can serve as a practical proxy for torque estimation with acceptable accuracy under laboratory operating conditions. Furthermore, the combination of experimental validation and system-level modeling enables deeper insight into the role of transmission losses, dynamic response, and measurement limitations, enhancing both educational and research value.

Table 1 summarizes how each solution maps to the cost drivers and outlines the residual risks.

### Impact and significance.

By eliminating these four cost drivers, our platform delivers functionality comparable to commercial systems at just 3% of the price ($370–$488 vs. $15,000+). More importantly, its transparency – students can inspect raw ADC counts before calibration and observe how physical principles translate to measurements – makes it an ideal teaching tool. All design files, firmware, and documentation are permanently archived under an open-source license (CERN OHL v2) with a DOI, inviting global adoption and adaptation.

**Table 1:** Direct mapping of cost drivers to solutions, cost impact, and residual risks.

Cost driver	Our solution	Cost eliminated	Residual risk
Dedicated torque transducer ($500–$4000)	Floating caliper + load cell ($3) + lever arm (100.0 mm) [17], [18]	$500–$4000	Alignment sensitivity, bearing friction, brake hysteresis
External electrical sensors ($200–$500)	Open-source ESC telemetry decoding (V/I/RPM/T) at 9600 baud [13]	$200–$500	Proprietary protocol, internal sensor bias
Proprietary DAQ ($500–$3000)	ESP32 ($5) + open-source firmware [15]	$500–$3000	Requires validation; no factory calibration
Specialized fabrication ($300–$800)	Off-the-shelf e-bike components [16]	$300–$800	Transmission losses, manual brake control
Maintenance ($300–$900/cycle)	Simple weight-based calibration procedure; documented maintenance schedule	Recurring cost avoided	Drift of low-cost load cell; pad wear

### 1.1 Sustainability and adaptability considerations

While this implementation uses the Votol EM-50 ESC, the design philosophy ensures adaptability:

• **Protocol documentation:** The complete telemetry protocol (packet structure, checksum, scaling) is documented in the repository.
• **Modular firmware:** Separate modules allow easy replacement of the Votol decoder with parsers for other ESC brands that offer serial telemetry.
• **Fallback mode:** Provision for external sensors is included in case telemetry is unavailable.
• **Industry standards:** The throttle input is standard 0–5 V analog, and mechanical interfaces follow common e-bike conventions.

The modular parser structure also facilitates future adaptation to other ESC brands that provide serial telemetry, such as Kelly and Sabvoton controllers.

## 2 Hardware description

### 2.1 System architecture overview

**Fig. 1:**
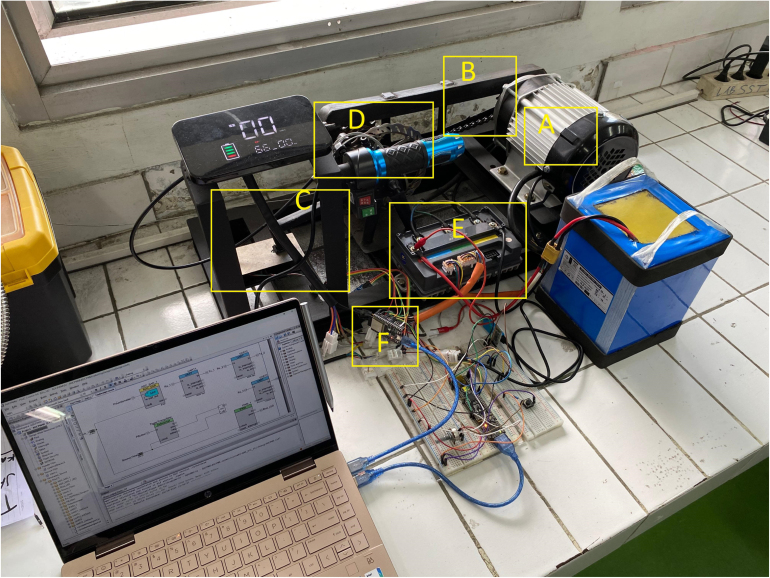
System architecture. (A) 1000 W BLDC motor, (B) chain transmission (14:22 ratio), (C) load shaft with brake disc, (D) floating brake assembly, (E) Votol EM-50 ESC with telemetry output, (F) ESP32-based control and data acquisition unit. **Important: The chain guard shown MUST be installed during all operations.**

The test bed integrates mechanical, electrical, and computational subsystems into a unified platform for BLDC motor evaluation. Fig. 1 shows the complete setup: a 1000 W mid-drive BLDC motor drives a load shaft via a chain transmission (14:22 ratio). A mechanical disc brake on the load shaft provides adjustable loading. The Votol EM-50 ESC controls the motor while streaming telemetry data. An ESP32 microcontroller generates throttle commands, acquires telemetry data, reads the torque sensor, and communicates with a host computer for data logging and visualization.

### 2.2 Component specifications

Table 2, Table 3, Table 4 detail the key components. For typical laboratory usage, significantly smaller battery capacities (e.g., 20–40 Ah) are sufficient, and the larger pack used here does not influence the measurement methodology.

**Table 2:** BLDC motor specifications (BM1418ZXF).

Parameter	Specification
Type	Brushless DC (BLDC), mid-drive
Rated Power	1000 W
Rated Voltage	48/60 V DC
Efficiency	>75%
Pole Pairs	14
Hall Sensors	Integrated (5-wire)
Weight	3.2 kg
Dimensions	140×180mm (diameter × length)

**Table 3:** Votol EM-50ESC specifications.

Parameter	Specification
Rated current	50 A continuous
Peak current	190 A (10 s)
Rated voltage	72 V DC max
Maximum power	3960 W
Efficiency	92%
Communication	Serial telemetry (TTL 5 V, 9600 baud)
Throttle input	0–5 V analog
Protection	Over-current, over-temperature, under-voltage
Waterproof rating	IP67

**Table 4:** LiFePO_4_ battery specifications (20S1P).

Parameter	Specification
Chemistry	Lithium Iron Phosphate (LiFePO_4_)
Configuration	20S1P (20 cells in series)
Nominal voltage	64 V (20×3.2 V)
Fully charged voltage	73 V (20×3.65 V)
Capacity	100 Ah
Energy	6.4 kWh
Rated continuous current	100 A (limited by BMS)
Peak current (10 s)	200 A
BMS	Built-in with balancing, over-current, over-voltage, under-voltage protection
Weight	≈25 kg
Dimensions	400×200×150mm (approx)

### 2.3 Measurement principles and theoretical background

#### 2.3.1 Electrical measurement via ESC telemetry

The Votol EM-50 ESC continuously monitors DC bus voltage (via an internal divider), DC bus current (Hall-effect), motor speed (hall sensor signals), and temperature (thermistor). These measurements are digitized and transmitted serially at 9600 baud. Decoding this data stream yields calibrated readings without any external sensors.

*Note: The current reported by telemetry is the DC bus current, not the phase current; this distinction is important when estimating torque from electrical parameters using the motor torque constant. Direct phase current measurement would require additional Hall-effect sensors, increasing cost and complexity, which contradicts the low-cost philosophy of this platform.*

**Limitations and error analysis:**

• **Proprietary protocol:** The protocol was reverse-engineered; modular firmware minimizes the impact of future firmware updates.
• **Accuracy dependency:** Validation (Section 7) shows a consistent positive bias in current (＋0.93% to ＋1.20%), likely due to factory calibration offset.
• **Error propagation:** The combined uncertainty in power (P=V×I) is
  (1) $$\frac{\Delta P}{P}=\sqrt{{\left(\frac{\Delta V}{V}\right)}^{2}+{\left(\frac{\Delta I}{I}\right)}^{2}}\approx \pm 1.23\text{\%}$$
  using worst-case ΔV/V=0.25% and ΔI/I=1.20%.

#### 2.3.2 System-level modeling

To provide a physics-based understanding of the system behavior, the BLDC motor is modeled using the dq-axis formulation [19]. The parameter set used here was obtained from experimental identification and validation conducted on the developed BLDC motor test bed and was then used for the HardwareX manuscript context.

The electrical dynamics are described by:

(2) $$v_{d}=Ri_{d}+L_{d}\frac{di_{d}}{dt}-\omega L_{q}i_{q}$$

(3) $$v_{q}=Ri_{q}+L_{q}\frac{di_{q}}{dt}+\omega \left(L_{d}i_{d}+\lambda_{pm}\right)$$

The electromagnetic torque is:

(4) $$T_{e}=\frac{3}{2}\frac{P}{2}\left(\lambda_{pm}i_{q}+\left(L_{d}-L_{q}\right)i_{d}i_{q}\right)$$

The mechanical dynamics are expressed as:

(5) $$J\frac{d\omega }{dt}=T_{e}-T_{L}-B\omega$$

For practical implementation, a simplified relationship is used:

(6) $$T_{e}\approx K_{t}I$$

where I is approximated by the DC bus current obtained from ESC telemetry.

The variables in Eqs. (1)–(5) are defined as follows: $v_{d}$ and $v_{q}$ are the direct- and quadrature-axis voltages (V); $i_{d}$ and $i_{q}$ are the direct- and quadrature-axis currents (A); R is the stator resistance (Ω); $L_{d}$ and $L_{q}$ are the direct- and quadrature-axis inductances (H); ω is the electrical angular speed (rad/s); $\lambda_{pm}$ is the permanent-magnet flux linkage (Wb); $T_{e}$ is the electromagnetic torque (N m); $T_{L}$ is the load torque (N m); J is the rotor inertia (kg m${}^{2}$); B is the viscous damping coefficient (N m s/rad); $K_{t}$ is the torque constant (N m/A); and I is the DC bus current obtained from ESC telemetry (A).

Table 5 summarizes the estimated model parameters used for system interpretation and validation.

These parameters are consistent with the experimentally validated motor model obtained from the developed test bed, where the dq-axis model produced a speed-response RMSE of 14.8 rpm under a 50% step-throttle experiment. This simplification enables low-cost implementation while maintaining acceptable accuracy for system-level analysis.

**Table 5:** Estimated BLDC motor model parameters.

Parameter	Value
R	0.5 Ω
$L_{d}$	0.8 mH
$L_{q}$	0.8 mH
$\lambda_{pm}$	0.12 Wb
J	0.002 kg m${}^{2}$
B	0.001 N m s/rad
P	8

#### 2.3.3 Mechanical torque measurement

**Fig. 2:**
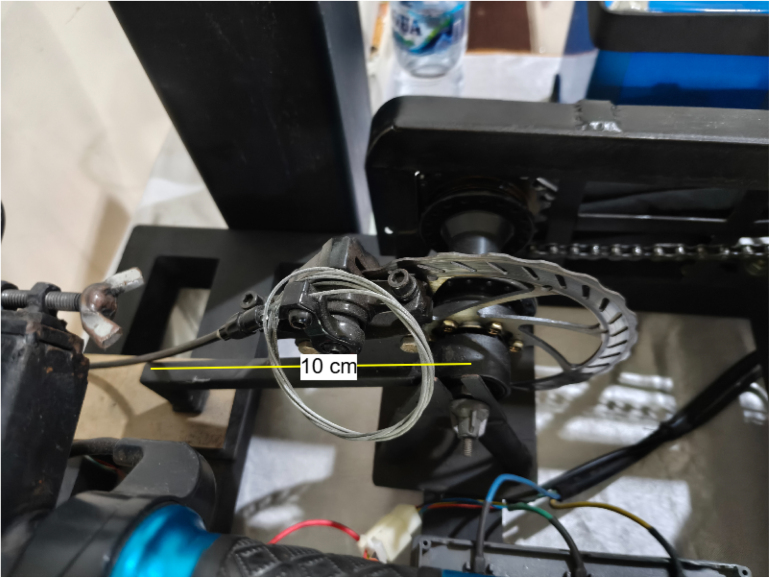
Floating caliper mechanism. The brake caliper mounts on a bearing-supported bracket, transferring reaction force through the lever arm to the load cell. The arm is inclined at 40° to the horizontal.

Torque is measured using a floating-caliper reaction method (Fig. 2). When braking force is applied, the caliper tries to rotate; this rotation is constrained by a lever arm (100.0 mm) connected to a 20 kg load cell. In our implementation, the torque arm is inclined at an angle of 40° relative to the horizontal plane to accommodate the brake caliper geometry. The load cell is mounted vertically, measuring the vertical component of the reaction force. Therefore, the torque at the load shaft is calculated as:

(7) $$T_{\text{load}}=F\times L\times \cos40{}^{\circ}=F\times 0.1\;\text{m}\times 0.7660=F\times 0.0766\;\text{m}$$

where F is the force measured by the load cell (N), L is the effective lever-arm length (m), $T_{\text{load}}$ is the load-shaft torque (N m), and GR is the dimensionless transmission ratio. The motor torque is then obtained by accounting for the transmission ratio (GR=22/14=1.57):

(8) $$T_{\text{motor}}=T_{\text{load}}/1.57$$

**Structural analysis:**

**Fig. 3:**
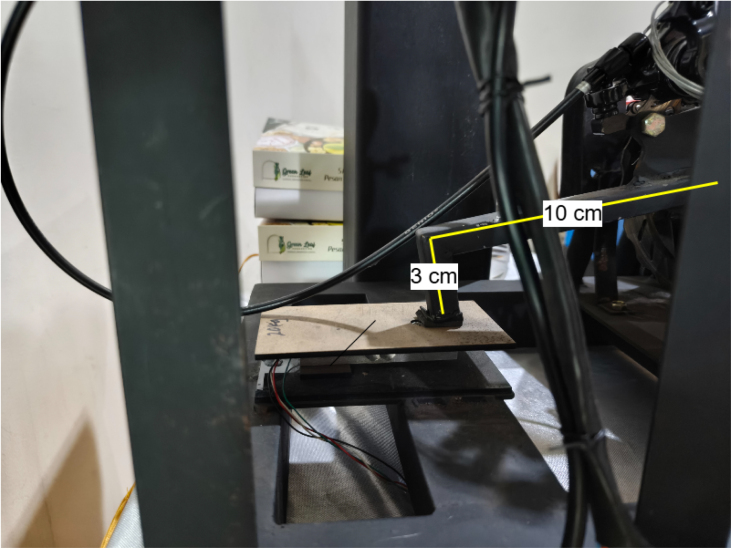
Torque measurement implementation. The load cell interfaces with an HX711 24-bit ADC, providing about 0.1 N resolution. The vertical mounting of the load cell is visible.

• **Alignment error:** A misalignment θ=±1° from the nominal 40° causes an error in cosθ of about 0.015%.
• **Lever arm deflection:** Under a maximum force of 100 N, the stainless-steel arm (cross-section 20×5mm) deflects by approximately 0.02 mm, changing the effective lever length by less than 0.02%.
• **Bearing friction:** The sealed ball bearings contribute less than 0.01 N m of friction torque, which is below 0.5% of the full-scale reading, as supported by the torque-arm implementation shown in Fig. 3.

The implemented torque-arm and load-cell arrangement is shown in Fig. 3.

### 2.4 Control and data acquisition system

The ESP32 DevKit C board (dual-core) handles:

• **Core 0:** Time-critical tasks: throttle signal generation (8-bit DAC) and telemetry packet decoding.
• **Core 1:** HX711 load cell sampling and serial communication with the host.

**Fig. 4:**
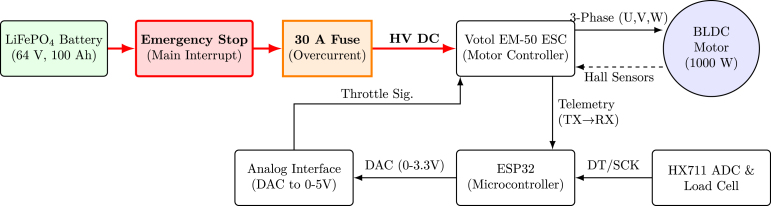
Electrical connections and safety diagram. Signal, power, and safety connections. The diagram explicitly highlights the main protection fuse (30 A) and the emergency stop circuit integrated into the high-voltage DC path for safe ESC operation.

Fig. 4 shows the complete wiring schematic, highlighting safety features such as a 30 A fuse, emergency stop, and voltage dividers for logic-level compatibility.

### 2.5 Comparative advantages

Table 6 contrasts our platform with commercial and other open-source dynamometers.

**Table 6:** Comparison with commercial and other open-source dynamometers.

Feature	Commercial (Magtrol)	Other Open-Source	This Work
Cost	$15,000+	$800–$2000	$370–$488
Torque sensing	Precision rotary	Often rotary	Load cell + lever (40° inclination)
Electrical sensing	External sensors	External sensors	ESC telemetry
Software	Proprietary	Various	Open-source Python/Arduino
Educational value	Black box	Variable	Transparent design
Safety features	Comprehensive	Variable	Multiple redundant systems

### 2.6 Technical drawing standards

Mechanical drawings follow ISO 128. Vector graphics (SVG) and high-resolution photographs (600 DPI) are provided in the repository.

### 2.7 Critical dimensions and tolerances

Table 7 lists the critical mechanical dimensions and tolerances required for accurate replication.

**Table 7:** Critical mechanical dimensions.

Component	Dimension	Tolerance
Base frame	600×400mm	±2mm
Motor mounting plate	150×100×5mm	±1mm
Shaft center distance	200 mm	±0.5mm
Torque arm length	100.0 mm	±0.1mm
Torque arm inclination	40°	±1°
Brake disc runout	–	<1mm

### 2.8 Software architecture

**Fig. 5:**
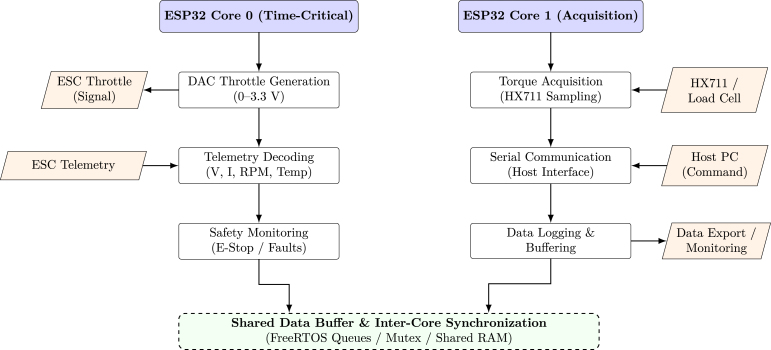
ESP32 dual-core firmware architecture. Task separation is implemented to ensure deterministic execution of time-critical motor-control functions on Core 0, while Core 1 handles data acquisition, communication, and logging tasks.

The firmware is distributed across the two ESP32 cores to improve determinism and responsiveness during operation. Core 0 handles time-critical functions such as DAC-based throttle generation, ESC telemetry decoding, and safety monitoring, while Core 1 handles HX711 torque acquisition, host communication, and data logging, as illustrated in Fig. 5.

## 3 Design files summary

We deliberately avoided designs requiring CNC machining. The platform uses standard steel stock, basic welding, and repurposed bicycle components. Documentation emphasizes photographic guidance over technical drawings, making it accessible to any workshop with conventional tools. All materials are released under CERN Open Hardware Licence Version 2 – Strongly Reciprocal [20]. The permanent archive is available at Zenodo: https://doi.org/10.5281/zenodo.20035899, while the actively maintained development repository is available at GitHub: https://github.com/uun3406/BLDC-TestBed-HardwareX (see Table 8).

**Table 8:** Design files summary.

File or folder	Description and format	Repository location
Wiring diagram	Clean system wiring diagram in PNG/SVG format showing the battery, ESC, BLDC motor, ESP32, HX711, load cell, throttle interface, host computer, and safety path.	hardware/wiring/
ESC connector pinout	Readable connector pinout reference in PDF/PNG/CSV format for the 2×8, 2×3, and 2 × 4 ESC connectors.	hardware/wiring/
ESP32 pin mapping	CSV mapping between ESP32 pins, ESC telemetry, throttle output, HX711 lines, and host interface.	hardware/wiring/
Frame dimensions	Mechanical dimension drawings in PDF/PNG format, including frame dimensions, shaft spacing, and fabrication tolerances.	hardware/mechanical/
Torque-arm dimensions	PDF/PNG documentation of lever-arm geometry and torque-arm inclination for floating-caliper torque measurement.	hardware/mechanical/
Assembly photographs	JPG/PNG photographs for frame cutting, welding, alignment, finishing, and component mounting.	media/photos/assembly/
Floating-caliper photographs	JPG/PNG photographs of the implemented floating-caliper torque reaction mechanism.	hardware/mechanical/
ESP32 firmware	INO/C++ firmware for throttle generation, telemetry acquisition, HX711 reading, logging, and calibration.	firmware/
Plotting scripts	Python scripts for regenerating model-validation, steady-state, dynamic-response, and torque-calibration plots.	scripts/
Experimental data	CSV/XLSX raw and processed telemetry, torque, steady-state, dynamic-response, and uncertainty-analysis data.	data/

## 4 Bills of material summary

Total cost ranges from USD 370 to 488, depending on regional pricing. The battery and motor account for about 60% of the total. Savings are possible through used components. Direct URLs for each component are provided below. All links were verified and accessible as of February 2025.

Table 9 provides the detailed bill of materials with direct sourcing links.

All URLs were last accessed in February 2025. For long-term archival, component sourcing details, firmware, documentation, and supplementary files are available in the Zenodo archive ( https://doi.org/10.5281/zenodo.20035899) and mirrored in the GitHub repository (https://github.com/uun3406/BLDC-TestBed-HardwareX).

**Table 9:** Detailed bill of materials with direct sourcing links.

ID	Component Description	Qty	Cost (USD)	Notes	Direct URL
MTR-01	1000 W BLDC mid-drive motor	1	110–130	BM1418ZXF	https://www.aliexpress.com/w/wholesale-mid-drive-motor.html
ESC-01	Votol EM-50controller with telemetry	1	40–50	Ensure telemetry enabled	https://www.aliexpress.com/w/wholesale-Votol-EM-50.html
MCU-01	ESP32 DevKit C development board	1	4–6	30-pin	https://www.amazon.it/dp/B0CT3BHJ4C
SNS-01	20 kg single-point load cell	1	2–3	Kitchen scale type	https://www.alibaba.com/product-detail/Load-Cell-1KG-5KG-10KG-20KG_1601088855762.html
SNS-02	HX711 24-bit ADC module	1	1–2	Load cell amplifier	https://www.aliexpress.com/w/wholesale-hx711-load-cell-sensor.html
BRK-01	Mechanical disc brake set	1	18–25	Mountain bike	https://www.amazon.sg/dp/B0BM4RKZMY
BRK-02	160 mm brake disc, 6-bolt	1	12–18	Standard	https://www.amazon.es/dp/B07WWT1QC3
MCH-01	Steel shaft, ∅20×300mm	1	8–12	Mild steel	https://www.tokopedia.com/berkatjayasakti/as-20-mm-plat-strip-besi-as-silinder-pejal-diameter-20mm-panjang-1-meter
MCH-02	Pillow block bearings, UCP204	2	6–8	With brackets	https://www.kramp.com/shop-de/en/p/pillow-block-bearing-complete--UCP204GP
MCH-03	Sprocket set 14T/22T with chain	1	10–15	Bicycle	https://www.aliexpress.com/w/wholesale-bicycle-sprocket-set.html
PWR-01	LiFePO_4_ battery pack, 64 V 100 Ah	1	200–250	20S1P, with BMS	https://www.optimumnano-china.com/lifepo4-battery/64v-100ah-lithium-battery-pack-long-life-for.html
FAB-01	Steel tubing 40×40×2mm	6 m	15–25	Frame	https://www.tokopedia.com/rumahbesiwf/hollow-40x40-2mm-hollow-hitam-4x4-2-0mm-hollow-besi-biasa-6-meter
FAB-02	Steel plate 150×100×5mm	1	3–5	Motor mount	https://www.tokopedia.com/cvn-steel/plat-besi-5mm-5-mm-x-50-cm-x-100-cm
HWD-01	Fasteners, bolts, nuts, washers	Set	8–12	M6, M8	https://www.tokopedia.com/rumahbaut/tag/baut-m6?page=1
WIR-01	Wiring, connectors, fuse holder	Set	5–10	16 AWG	https://www.aliexpress.com/w/wholesale-16awg-wire.html
ENCL-01	Project enclosure	1	5–8	Electronics	https://www.aliexpress.com/w/wholesale-project-enclosure.html

**Total cost range**			**370–488**		

## 5 Build instructions

### 5.1 Safety-first approach

Before starting, ensure proper personal protective equipment (safety glasses, welding mask, gloves) and adequate ventilation for welding and battery handling. General workshop safety practices must be followed.

To further assist in the replication process, supplementary videos demonstrating the complete assembly, steady-state operation, dynamic load testing, and emergency-stop behavior are available in the Zenodo archive and mirrored in the GitHub repository.

### 5.2 Step-by-step assembly procedure

The complete assembly can be summarized as follows:

1. Construct the steel frame according to the specified dimensions and tolerances.
2. Install the shaft and bearing system and verify rotational freedom.
3. Mount the BLDC motor and align the sprocket-chain transmission.
4. Install the brake disc and floating-caliper assembly on the load shaft.
5. Attach the torque arm at 40° and mount the load cell vertically.
6. Connect the HX711 module to the ESP32 and verify sensor output.
7. Wire the ESC, battery, emergency stop, and fuse-protected power path.
8. Integrate the ESP32 throttle, telemetry, and host-communication interfaces.
9. Verify chain guard installation, insulation, and emergency stop functionality.
10. Perform torque calibration, throttle calibration, and initial no-load startup testing.

### 5.3 Mechanical assembly sequence

**Fig. 7:**
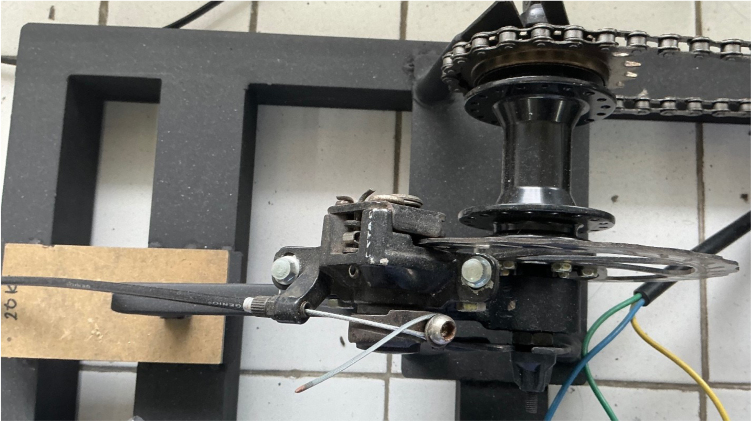
Implemented floating caliper torque reaction mechanism. The mechanical disc brake caliper is mounted on a pivoting bracket that allows limited rotational freedom. When braking torque is applied to the disc mounted on the load shaft, the caliper housing reacts against the support arm, transmitting force to the torque measurement linkage. The brake cable provides manual load adjustment, while the reaction arm transfers the braking force to the load cell for torque calculation based on the calibrated lever arm length (100.0 mm).

Frame construction, shaft/transmission assembly, and floating caliper fabrication are illustrated in Fig. 6, Fig. 7. Ensure the torque arm is welded at the correct 40° angle using a protractor or angle gauge. Supplementary videos demonstrating the step-by-step assembly process and system operation are available in the Zenodo archive and mirrored in the GitHub repository.

Fig. 6Mechanical frame fabrication and assembly process. (a) Cutting of 40 × 40 × 2 mm steel hollow sections. (b) Tack welding and preliminary alignment verification. (c) Full welding of the structural frame to ensure shaft center distance tolerance (±0.5mm). (d) Surface finishing and preparation for component mounting.Fig. 6

**(a):**
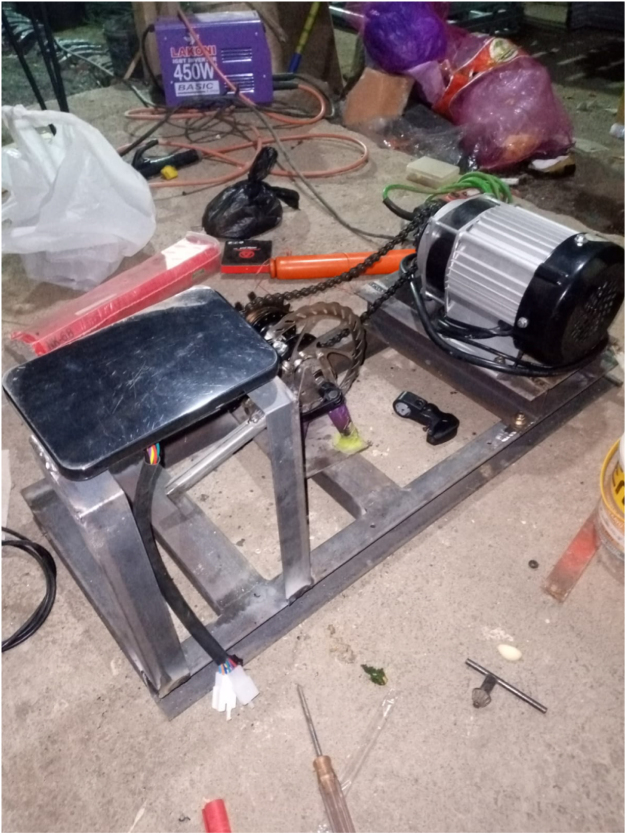

**(b):**
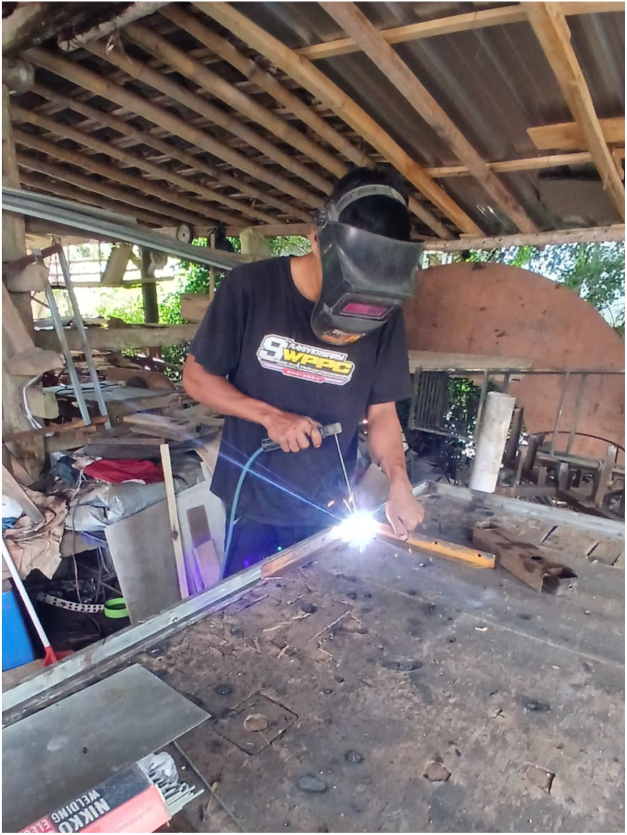

**(c):**
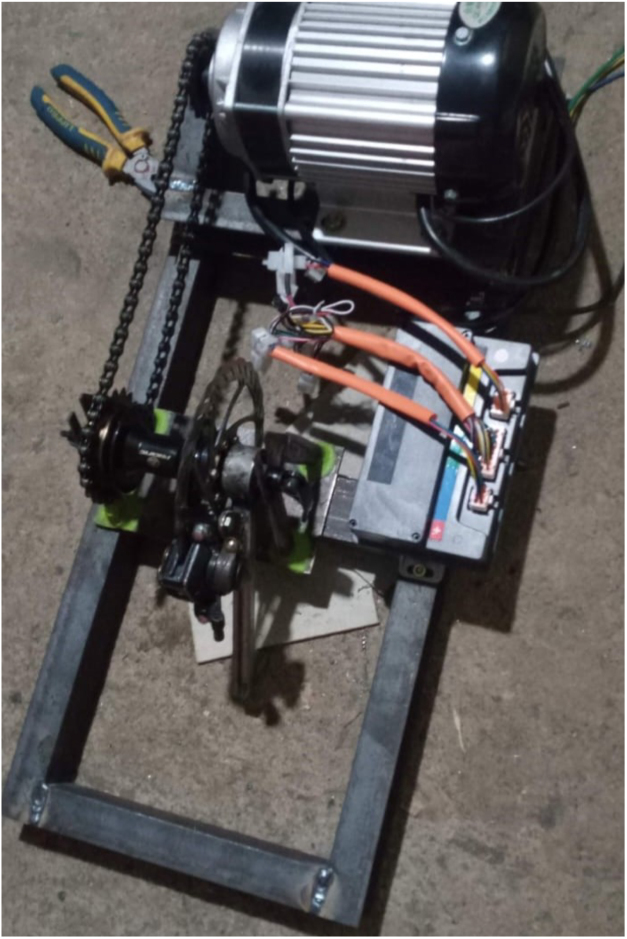

**(d):**
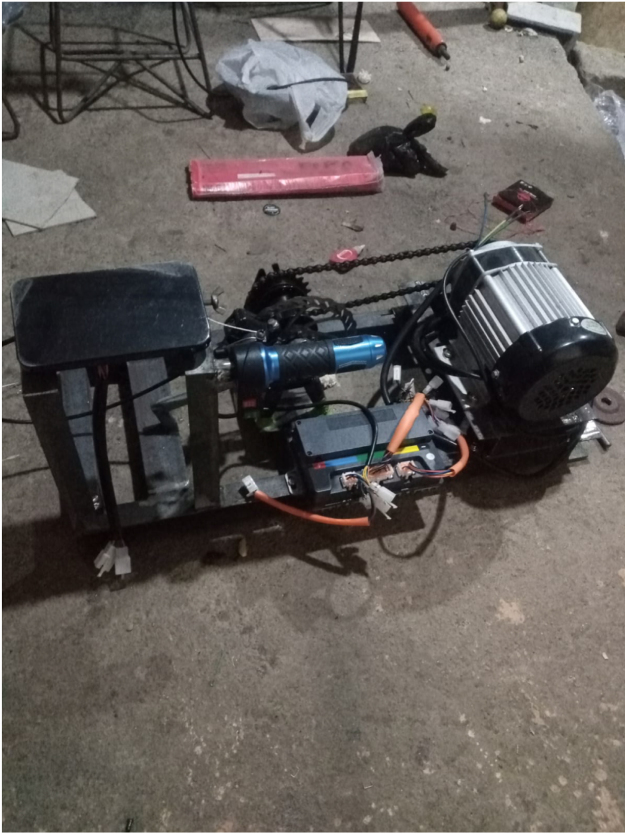

### 5.4 Electrical assembly

**Fig. 8:**
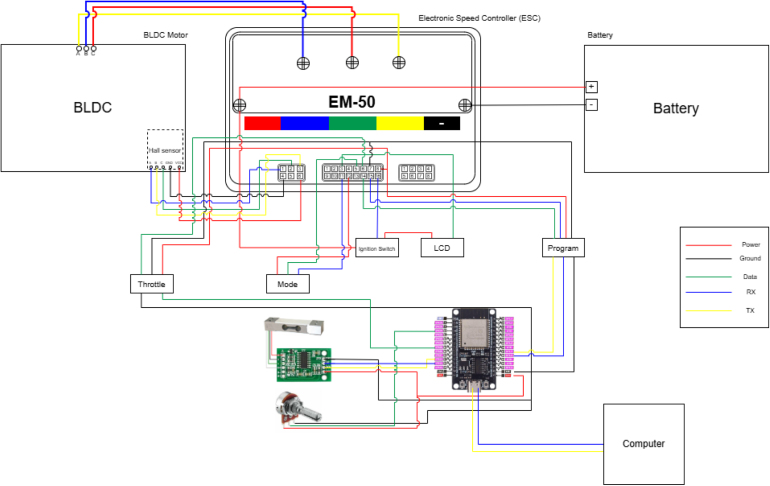
Complete system wiring diagram of the BLDC motor test bed. The LiFePO_4_ battery supplies high-voltage DC power to the Votol EM-50 ESC, which drives the three-phase BLDC motor (U–V–W). Hall sensor lines provide rotor position feedback to the ESC. The ESP32 microcontroller interfaces with (1) the ESC telemetry output through TTL serial communication, (2) the throttle input through DAC-based analog control and scaling, and (3) the load cell through the HX711 24-bit ADC module for torque measurement. The diagram also shows the main power path, control interface, telemetry lines, emergency-stop path, and low-voltage signal connections required for safe operation.

The complete electrical interconnection of the system is shown in Fig. 8. This detailed physical wiring diagram complements the conceptual safety and control schematic (Fig. 4) by providing implementation-level clarity. The diagram illustrates the full power, control, sensing, and communication wiring between the battery pack, ESC, BLDC motor, load cell module, throttle interface, and ESP32 microcontroller. To improve readability, the small embedded connector table previously placed inside the wiring diagram has been removed. The practical system connections are summarized separately in Table 10, while the ESC connector pin definitions are provided as separate LaTeX tables in Table 11, Table 12, Table 13. All connections must be verified carefully before initial power-up.

**Table 10:** Key wiring connections between ESC, ESP32, and sensors.

Source	Destination	Function
Battery positive	ESC power input	Main DC supply to ESC
Battery negative	ESC return	Main DC return path
ESC phase U/V/W	BLDC motor U/V/W	Three-phase motor drive output
Motor hall lines	ESC hall input	Rotor position feedback
ESP32 DAC output	ESC throttle input (via scaling stage)	Analog throttle command
ESC telemetry TX	ESP32 serial RX	Real-time telemetry acquisition
HX711 DT/SCK	ESP32 GPIO pins	Torque signal acquisition
Emergency stop switch	Main power interrupt/enable path	Safety shutdown
Fuse holder	Battery-ESC positive line	Overcurrent protection
ESP32 USB/serial	Host computer	Monitoring, logging, and control

**Table 11:** ESC 2 × 8 connector pinout.

Port	Function definition	Code	Voltage
1	Throttle power		5 V
2	Throttle GND		0 V
3	Throttle signal		0–5 V
4	3 gear	PA0	0–5 V
5	Repair/safe boost	PC15	0–5 V
6	P gear	PC14	0–5 V
7	Line output	PB9	0–5 V
8	High brake	PB5	0–12 V
9	Electric door lock		40 V–B+
10	UART-TX		0–5 V
11	UART-RX		0–5 V
12	R gear	PA11	0–5 V
13	1 gear	PB2	0–5 V
14	GND		0 V
15	S gear	PB3	0–5 V
16	Low brake	PB4	0–5 V

**Table 12:** ESC 2 × 3 hall connector pinout.

Port	Function definition	Voltage
1	Hall signal A	0–5 V
2	Hall signal B	0–5 V
3	Hall signal C	0–5 V
4	Hall power	5 V
5	Motor temp signal	0–5 V
6	Hall GND	0 V

**Table 13:** ESC 2 × 4 program and anti-theft connector pinout.

Port	Function definition	Code	Voltage
1	GND		0 V
2	JTCK		0–5 V
3	SWD		0–5 V
4	5 V input		0–5 V
5	Anti-theft power		B+
6	Anti-theft electric door lock		40 V–B+
7	Push signal		0 V–B+
8	Anti-theft signal	PA12	0–5 V

Note: connector labels and voltage ranges follow the ESC wiring documentation used in this build.

### 5.5 Pre-operation verification

**Fig. 9:**
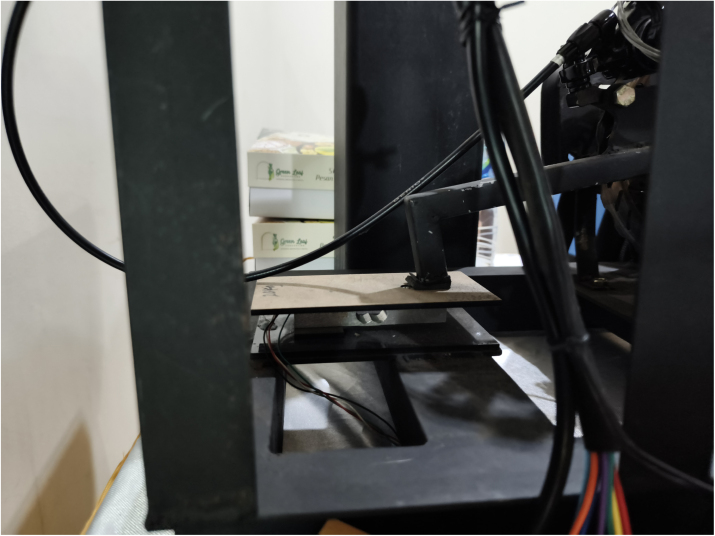
Load cell installation for torque measurement. The single-point load cell is mounted vertically between two steel plates fixed to the frame. The torque reaction arm transfers braking force downward onto the upper plate, generating a compressive load on the sensor. The load cell output is routed to the HX711 24-bit ADC module for force acquisition. Proper alignment ensures that only vertical force components are measured, minimizing lateral loading and measurement error.

Table 14 provides the pre-operation verification procedure to ensure safe and repeatable operation. The practical load-cell installation used for torque measurement is shown in Fig. 9.

**Table 14:** Pre-operation checklist.

Check	Procedure	Acceptance
Mechanical fasteners	Torque check all bolts	No movement
Electrical insulation	Continuity test, 500 V megger	>1 MΩ
Rotational freedom	Manual shaft rotation	Smooth, no binding
Chain guard	Verify complete installation	Fully enclosed
Emergency stop	Button press	Power interruption
Ground continuity	Resistance to earth	<1Ω
Battery safety	Check for damage/swelling	No defects
Torque arm angle	Measure with digital protractor	40°±1°

## 6 Operation instructions

### 6.1 Initial setup and calibration

#### 6.1.1 Load cell installation and torque calibration

1. Zero adjustment: with the brake fully released, execute the software zero command. The load cell should read 0±0.1 N.
2. Primary calibration: hang a certified 2.000 kg mass at the end of the torque arm (100.0 mm). The applied torque is mgLcos40°=2.000×9.80665×0.1×0.7660=1.502 N m. Enter this value; the software computes the calibration factor.
3. Verification: repeat with a 5.000 kg mass (3.755 N m). The error should be less than 1%.
4. Hysteresis check: remove the mass and verify that the reading returns to zero within 0.2% of full scale.

#### 6.1.2 Throttle calibration

To improve reproducibility, throttle calibration must be performed before operation.

1. Disconnect the motor phases for safety.
2. Run the throttle calibration routine in firmware.
3. Verify that the ESP32 DAC output spans 0.0 to 3.3 V, corresponding to 0%–100% digital throttle command.
4. Verify that the external voltage divider or analog adaptation stage scales this DAC output to 0.0–5.0 V for the ESC throttle input.
5. Confirm that the host monitoring interface reports the same 0%–100% throttle range.

In this architecture, the DAC does not directly generate 5 V. Instead, the microcontroller produces a calibrated analog control level that is subsequently matched to the ESC throttle input range. This clarification is important because the ESC expects a standard 0–5 V analog input.

### 6.2 ESC telemetry data structure

**Fig. 10:**
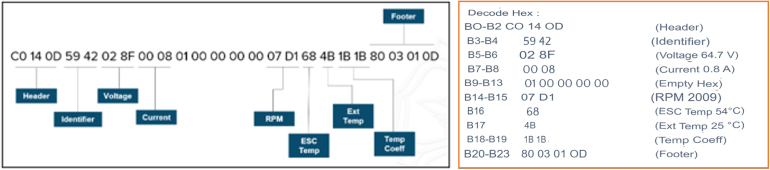
ESC telemetry frame structure and hexadecimal decoding. The ESC transmits a 24-byte serial data packet at 9600 baud. Bytes B0–B2 represent the header (C0 14 0D), followed by an identifier (B3–B4). Electrical and operational parameters are encoded in fixed byte positions: B5–B6 (DC bus voltage), B7–B8 (current), B14–B15 (motor speed in RPM), B16 (ESC internal temperature), B17 (external temperature), and B18–B19 (temperature coefficient). The frame terminates with a four-byte footer (B20–B23). The example shown corresponds to 64.7 V, 0.8 A, 2009 RPM, ESC temperature 54°C, and external temperature 25°C.

Fig. 10 illustrates the hexadecimal data frame transmitted by the ESC telemetry interface and its decoded parameter mapping. The scaling factors are: DC bus voltage = raw/10 (V), current = raw/10 (A), RPM = (high byte ≪ 8) | low byte, and temperature = raw (with offset as per datasheet). Invalid frames are discarded by the firmware.

### 6.3 Safety protocols

**CRITICAL SAFETY WARNINGS:**

• **Lithium battery hazard:** The 64 V LiFePO_4_ pack stores significant energy. Never short-circuit, puncture, or expose it to high temperatures.
• **Rotating chain guard:** The chain guard shown in Fig. 1 MUST be installed during all operations.
• **High voltage:** The ESC operates at up to 73 V DC. Insulate all connections and use insulated tools.
• **Thermal monitoring:** Continuously monitor ESC temperature via telemetry. Automatic shutdown occurs at 85°C; manual intervention is required if temperatures exceed 75°C during sustained operation.
• **Emergency procedures:** All operators must know the location of the emergency stop button. Keep a Class C fire extinguisher accessible.
• **Two-person rule:** Initial testing and calibration should be conducted with two qualified personnel, one dedicated to safety monitoring.

### 6.4 Shutdown and maintenance

#### 6.4.1 Normal shutdown

Reduce throttle to zero, wait for complete stop, release the brake, and disconnect the battery.

#### 6.4.2 Regular maintenance

Table 15 summarizes the maintenance schedule used during long-term operation.

**Table 15:** Maintenance schedule.

Interval	Task	Acceptance
Daily	Bolt tightness	No movement
Weekly	Chain lubrication	Smooth operation
Monthly	Brake pad inspection	>1mm remaining
Monthly	Battery health check (voltage balance, no swelling)	Cells within 50 mV, no deformation
Semester	Full calibration	Within spec
Annual	Bearing inspection	Smooth rotation, no play

## 7 Validation and characterization

### 7.1 Experimental methodology

All validation tests were performed under controlled conditions (25±2°C, 50±10% RH). Reference instruments included:

• Fluke 87V multimeter (voltage verification)
• Fluke i400 current clamp + Fluke 87V (current verification)
• Ono Sokki HT-5500 optical tachometer (speed validation)
• Mettler Toledo certified weights (torque calibration)
• Fluke Ti400 thermal imager (temperature correlation)

Unless otherwise noted, validation measurements were taken under steady-state operating conditions after the manually applied brake load had stabilized. The representative loading states used throughout the validation section correspond to approximately 25%, 50%, and 75% manual brake application, established by prior calibration of load-cell force against the floating-caliper torque arm. Dynamic tests were performed separately using step application and release of the brake cable.

### 7.2 Telemetry accuracy assessment

Table 16 compares ESC telemetry readings with reference measurements across the operational range.

Telemetry errors are below 3% for electrical parameters and 5% for temperature, well within the needs of undergraduate laboratories. The consistent positive bias in current (0.93–1.20%) suggests a systematic offset in the ESC internal calibration.

**Table 16:** Telemetry accuracy (n=20).

Condition	Telemetry	Reference	Abs Error	Rel Error
**Voltage (V)**				
No load	52.3	52.41	−0.11	−0.21%
Full load	48.7	48.82	−0.12	−0.25%

**Current (A)**				
25% load	4.2	4.15	+0.05	+1.20%
50% load	8.7	8.62	+0.08	+0.93%
75% load	13.1	12.95	+0.15	+1.16%

**Speed (RPM)**				
1000 setpoint	1005	1002	+3	+0.30%
2000 setpoint	1992	1995	−3	−0.15%
3000 setpoint	2988	2991	−3	−0.10%

**Temperature** (°C)				
After 5 min	42	40.5	+1.5	+3.70%
After 10 min	58	56.2	+1.8	+3.20%

### 7.3 Torque measurement validation

#### 7.3.1 Direct calibration

Using NIST-traceable calibration weights, we established the relationship between load cell output and applied torque [18]. The calibration factor is defined as:

(9) $$\text{Calibration factor}=\frac{\text{Applied Torque (N m)}}{\text{ADC Counts}}=\frac{mgL\cos40{}^{\circ}}{\text{ADC Counts}}$$

With L=0.1 m, cos40°=0.7660, and using a 2.000 kg mass, the applied torque is 1.502 N m. The corresponding ADC count yielded a factor of $2.184\times 10^{-5}$ N m/count. Linearity across the 0–10 N m range yielded $R^{2}=0.9993$, confirming excellent linear response.

#### 7.3.2 Cross-verification with electrical method

Motor torque was independently estimated from electrical parameters using the manufacturer-specified torque constant ($K_{t}=0.32$ N m/A). Note that $K_{t}$ is typically defined for phase current; here we use DC bus current as a proxy:

(10) $$T_{\text{electrical}}=K_{t}\times I_{\text{phase}}\approx K_{t}\times I_{\text{DC}}$$

This approximation is valid under steady-state conditions where the relationship between DC bus current and q-axis current remains approximately proportional, although deviations may occur under highly dynamic PWM operation or significant low-speed ripple.

Table 17 compares mechanical and electrical torque estimation.

The increasing discrepancy at higher loads can be explained using the system-level model. According to the mechanical equation,

(11) $$J\frac{d\omega }{dt}=T_{e}-T_{L}-B\omega$$

the measured torque includes loss components such as chain transmission losses, bearing friction, and mechanical damping. Additionally, the use of DC bus current instead of phase current introduces approximation error. Therefore, the observed 3%–5% deviation is dominated by system-level losses rather than measurement inaccuracies.

**Table 17:** Torque comparison (n=10).

Load	Mechanical	Electrical	Difference
Light (25%)	1.18±0.03	1.15±0.04	+2.6%
Medium (50%)	3.76±0.04	3.65±0.05	+3.0%
Heavy (75%)	6.52±0.05	6.21±0.06	+5.0%

### 7.4 Uncertainty budget analysis

Following ISO/IEC Guide 98-3 (GUM), we performed a comprehensive uncertainty analysis for torque measurement at a typical operating point of 5 N m. Table 18 summarizes the uncertainty budget.

This analysis confirms that the measurement system itself contributes less than 0.5% uncertainty.

**Table 18:** Uncertainty budget for torque measurement at 5 N m.

Source	Type	Value	Contribution
Load cell linearity	B	±0.1% FS	0.058%
HX711 quantization	B	±0.01%	0.006%
Lever arm length	B	±0.1mm (0.1%)	0.058%
Calibration factor (from mass, length, and repeatability)	B	±0.15%	0.087%
Temperature drift	B	±0.05%/10°C	0.029%
Repeatability	A	±0.3% (n=10)	0.173%

Combined std uncertainty			0.214%
Expanded uncertainty (k=2, 95% confidence)			0.428%

### 7.5 Model validation

The validity of the adopted system model was evaluated by comparing simulated and experimental speed responses under a step-throttle input corresponding to approximately 50% throttle, with a target speed near 2000 RPM. This validation procedure and the resulting RMSE are consistent with the experimentally validated system model, which used the same motor parameter set (R=0.5Ω, $L_{d}=L_{q}=0.8$ mH, $\lambda_{pm}=0.12$ Wb, J=0.002 kg m${}^{2}$, B=0.001 N m s/rad, P=8) and showed close agreement between simulated and averaged experimental responses. The validation test was performed under stabilized operating conditions, and no additional manual brake adjustment was introduced during the transient response window. Five repeated experiments were conducted, and the pointwise average speed response was used for comparison with the model.

**Fig. 11:**
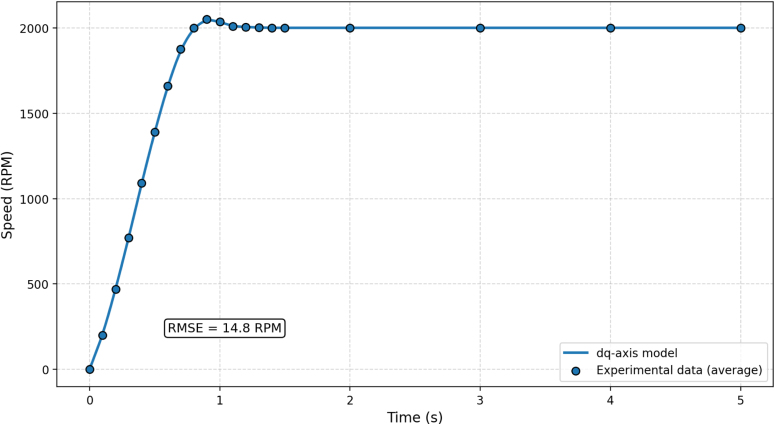
Model versus experimental speed response under a step input. The dq-axis-based model reproduces the main transient and steady-state features of the measured response. The resulting RMSE is 14.8 rpm, supporting the use of the model for interpretation of system behavior and controller assessment.

The measured speed response was consistent with the dynamics predicted by the dq-axis-based motor model. The model reproduced the main acceleration and settling trends observed experimentally, indicating that the simplified formulation is sufficient for system-level interpretation of the platform behavior. Model validation on the same experimental platform yielded an RMSE of approximately 14.8 rpm between simulated and experimental speed response, indicating that the model adequately captures the system dynamics and is suitable for interpretation of test-bench behavior. The comparison between the simulated and experimental speed responses is shown in Fig. 11.

### 7.6 Steady-state performance characterization

Fig. 12 shows typical steady-state performance at three operating speeds.

Key observations:Fig. 12Steady-state performance at 1000, 2000, and 3000 RPM. (a) Motor current stability under constant load. (b) Speed regulation performance. Data were collected over 30-second intervals at constant load, and all axes are labeled with physical units.Fig. 12

**(a):**
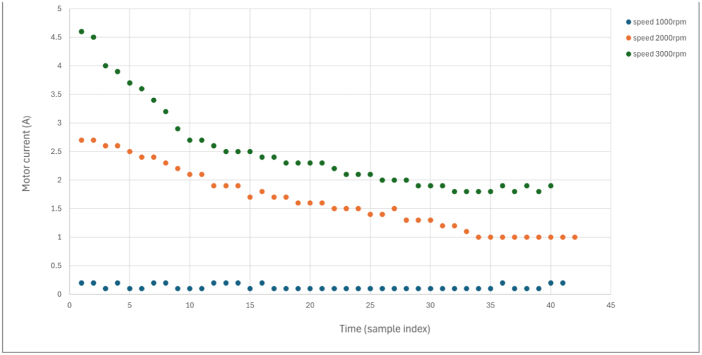

**(b):**
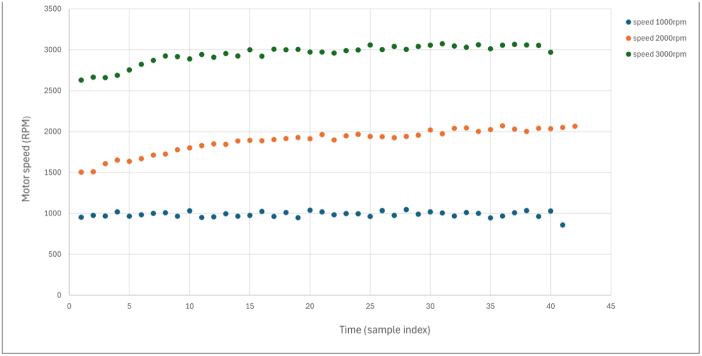

• Speed regulation remains within ±1% of setpoint.
• Current measurements show minimal drift (<2%) during 5-minute operation.
• Temperature rise follows a predictable thermal time constant (∼3 min to steady state).
• Results are repeatable across multiple test sessions (coefficient of variation <3%).

### 7.7 Dynamic response analysis

Transient response to step load changes (Fig. 13) reveals the dynamic capability of the platform under more realistic laboratory loading conditions. The dynamic test was conducted by operating the motor at a fixed setpoint and then applying and releasing the manual brake in a repeatable step-like manner, producing an effective load step of approximately 50% of the calibrated braking range. This test evaluates transient behavior in addition to steady-state performance.

Dynamic characteristics:Fig. 13Dynamic response to step load application and removal. (a) Current response to a 50% load step. (b) Speed dip and recovery. (c) Torque response during transient loading. Data were sampled at 10 Hz, and all axes are labeled with physical units.Fig. 13

**(a):**
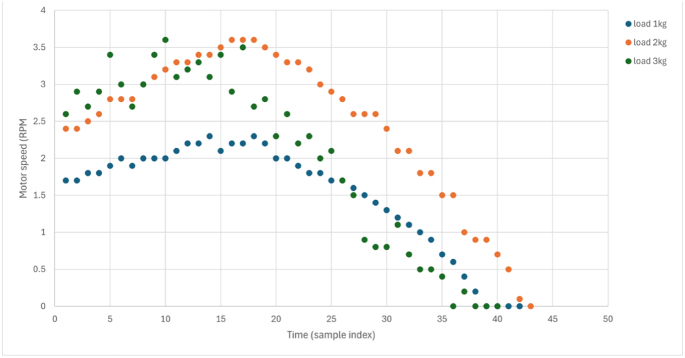

**(b):**
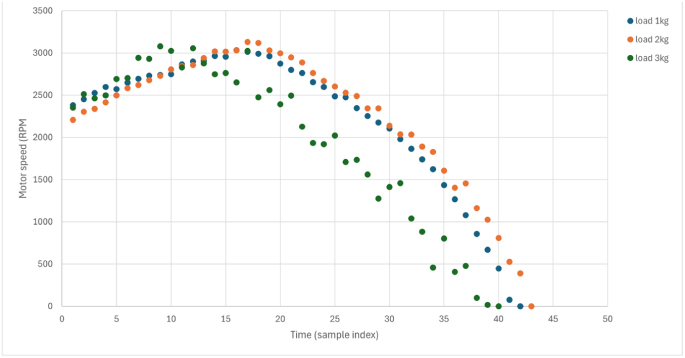

**(c):**
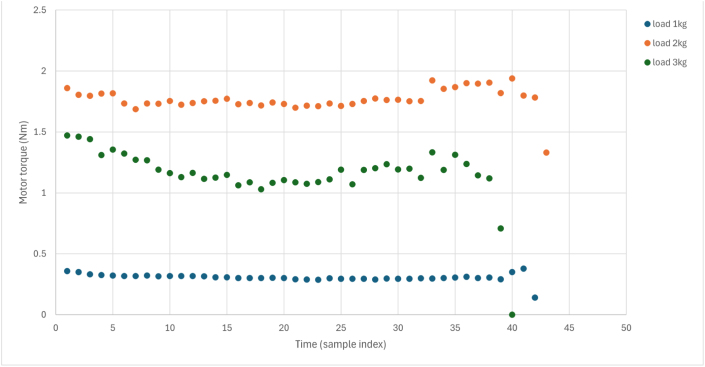

• Current response time: 0.4 s to reach 90% of steady-state.
• Speed recovery time: 1.2 s to return within 2% of setpoint.
• Torque measurement latency: <0.2 s (limited by the 10 Hz logging rate).
• No oscillation or instability, indicating a well-damped system.

The observed transient response closely follows the behavior predicted by the simplified mechanical model. The rise-time and recovery characteristics are consistent with the identified system inertia and damping parameters, confirming that the dominant dynamics are governed by mechanical load interaction rather than control instability. These responses show that the platform is suitable not only for steady-state analysis but also for dynamic performance evaluation, directly addressing a key limitation noted in prior low-cost laboratory systems. Supplementary videos documenting this dynamic testing are available in the Zenodo archive and mirrored in the GitHub repository.

### 7.8 Educational implementation results

The platform has been used in undergraduate laboratories at a university in Indonesia, supporting the modules listed in Table 19. An anonymous survey was conducted for educational improvement purposes only.

Student feedback (n=42) indicated:

**Table 19:** Educational laboratory modules implemented.

Module	Learning objectives
Motor performance mapping	Generate torque-speed curves, identify operating points
Efficiency analysis	Calculate efficiency vs. load, find peak efficiency
Dynamic response study	Observe transient behavior, measure response times
Control system concepts	Understand ESC feedback control, observe regulation
Instrumentation principles	Sensor interfacing, data acquisition, uncertainty analysis
Safety engineering	Risk assessment, safety protocol development

• 92% agreed the platform enhanced their understanding of motor characteristics.
• 88% found the open design helped them grasp measurement principles.
• 95% considered the hands-on experience valuable for career preparation.
• 96% rated the safety instructions as clear and comprehensive.

### 7.9 Limitations and future improvements

Table 20 summarizes current limitations and potential enhancements.

**Table 20:** Current limitations and potential enhancements.

Limitation	Impact	Potential Solution
Manual brake control	Limited precision in load application	Servo-actuated brake with closed-loop control
Thermal capacity	30-minute continuous operation limit	Enhanced cooling or higher-capacity brake
Speed measurement	RPM-only (no position feedback)	Add encoder for position control
Single motor type	Optimized for mid-drive BLDC	Modular mount for different motor types
ESC dependency	Votol-specific telemetry	Generalized telemetry decoder library

### 7.10 Long-term reliability assessment

After six months of academic use (approximately 50 operational hours):

• No mechanical failures or component replacements.
• Measurement accuracy remained within original specifications.
• Minor maintenance included chain tension adjustment (once) and brake pad replacement (once).
• Software remained stable with no crashes or reboots.
• Student-induced issues were resolved through documented troubleshooting.
• Safety systems (emergency stop) were tested weekly and functioned correctly.

### 7.11 Discussion

The integration of ESC telemetry with system-level modeling provides a clearer scientific contribution compared with existing low-cost dynamometer platforms. While conventional systems rely on direct phase current measurement, this study shows that DC bus current can serve as a practical proxy with acceptable error margins for educational and laboratory characterization purposes.

A practical limitation of the present platform is the use of manual brake actuation, which reduces the precision and repeatability of load application compared with servo-controlled or fully automated dynamometer systems. For this reason, the platform is positioned as a low-cost educational and laboratory characterization system rather than a metrology-grade dynamometer. Nevertheless, the observed repeatability in steady-state operation and the consistency of transient trends indicate that the system remains suitable for instructional experiments and comparative performance evaluation.

The results highlight that:

• Measurement deviations are dominated by system losses rather than sensor limitations.
• Modeling significantly improves interpretability of experimental data.
• The system bridges the gap between theoretical motor modeling and real-world laboratory implementation.

The system is intended for educational and laboratory characterization purposes and is not designed to replace high-precision industrial dynamometers.

## CRediT authorship contribution statement

**Uun Triyas Yuni Kurniawan:** Writing – original draft, Visualization, Validation, Software, Methodology, Investigation, Formal analysis, Data curation, Conceptualization. **Faridah:** Writing – review & editing, Supervision, Resources, Project administration, Funding acquisition. **Sunarno:** Writing – review & editing, Validation, Supervision, Resources, Methodology.

## Ethics statements

No human participants, animal subjects, or biological materials were involved. Research was conducted according to institutional safety guidelines.

## Declaration of competing interest

The authors declare that they have no known competing financial interests or personal relationships that could have appeared to influence the work reported in this paper.

## Data Availability

All files required to evaluate, reproduce, and inspect the hardware platform are publicly accessible. All design files, source code, documentation, and materials are publicly available under an open-source license. The supplementary videos are available in the Zenodo archive and mirrored in the GitHub repository. The permanent archived version used for scholarly record is hosted on Zenodo: https://doi.org/10.5281/zenodo.20035899 The actively maintained development repository is available on GitHub: https://github.com/uun3406/BLDC-TestBed-HardwareX.
